# The Synthetic Flavonoid Derivative GL-V9 Induces Apoptosis and Autophagy in Cutaneous Squamous Cell Carcinoma via Suppressing AKT-Regulated HK2 and mTOR Signals

**DOI:** 10.3390/molecules25215033

**Published:** 2020-10-30

**Authors:** Yejin Zhu, Mengdi Liu, Jingyue Yao, Qinglong Guo, Libin Wei

**Affiliations:** 1 State Key Laboratory of Natural Medicines, Jiangsu Key Laboratory of Carcinogenesis and Intervention, China Pharmaceutical University, 24 Tongjiaxiang, Nanjing 210009, China; yejin_zhu@foxmail.com (Y.Z.); mmdlmd666@163.com (M.L.); yaojingyue1114@163.com (J.Y.); 2 School of Medicine & Holistic Integrative Medicine, Nanjing University of Chinese Medicine, 138 Xianlin Rd, Nanjing 210033, China

**Keywords:** GL-V9, cutaneous squamous-cell carcinoma, apoptosis, autophagy, AKT, HK2

## Abstract

Cutaneous squamous-cell carcinoma (cSCC) is one of most common type of non-black skin cancer. The malignancy degree and the death risk of cSCC patients are significantly higher than basal cell carcinoma patients. GL-V9 is a synthesized flavonoid derived from natural active ingredient wogonin and shows potent growth inhibitory effects in liver and breast cancer cells. In this study, we investigated the anti-cSCC effect and the underlying mechanism of GL-V9. The results showed that GL-V9 induced both apoptosis and autophagy in human cSCC cell line A431 cells, and prevented the growth progression of chemical induced primary skin cancer in mice. Metabolomics assay showed that GL-V9 potentially affected mitochondrial function, inhibiting glucose metabolism and Warburg effect. Further mechanism studies demonstrated that AKT played important roles in the anti-cSCC effect of GL-V9. On one hand, GL-V9 suppressed AKT-modulated mitochondrial localization of HK2 and promoted the protein degradation of HK2, resulting in cell apoptosis and glycolytic inhibition. On the other hand, GL-V9 induced autophagy via inhibiting Akt/mTOR pathway. Interestingly, though the autophagy induced by GL-V9 potentially antagonized its effect of apoptosis induction, the anti-cSCC effect of GL-V9 was not diluted. All above, our studies suggest that GL-V9 is a potent candidate for cSCC treatment.

## 1. Introduction

Cutaneous squamous cell carcinoma (cSCC), originating from the epidermis or adnexal keratinocytes, is one of the highest incidence of non-melanocytic skin carcinoma, only second to basal cell carcinoma. Because cSCC is a kind of highly metastatic malignant tumors, the death risk of cSCC patients is much higher than the patients with basal cell carcinoma [1]. Improvements in treatment of cSCC could be by means of novel agents targeting the signaling pathways that facilitate cancer cell growth and survival [2].

In recent years, a mushrooming of attention and expectations in cancer treatment have been paid to the studies of the active ingredients from natural products. Flavonoid wogonin is extracted from the traditional Chinese medicine *Scutellaria baicalensis* with multiple of pharmacological effects, including anticancer, antioxidation, anti-inflammation, and so on [3]. Although wogonin shows great development value in pharmacological action, poor water-solubility, and pharmacokinetic characteristics increase the difficulty to develop wogonin as a drug [4]. GL-V9 is derivative from wogonin, which is improved in druggability and shows potent anti-tumor effect in several of cancer cells via inducing apoptosis and cell cycle arrest, as well as inhibiting invasion [5,6,7,8]. However, the anticancer effects of GL-V9 on cSCC are rarely reported.

Tumor cells prefer to choose glycolysis to produce energy even under well-oxygenated conditions, instead normal cells will choose oxidative phosphorylation pathway. This phenomenon is called the Warburg effect, also known as aerobic glycolysis [9]. Previous study shows that GL-V9 inhibits glycolysis of breast cancer via disrupting mitochondrial binding of hexokinase II (HK2) [10]. Hexokinase is the first rate-limiting enzyme of glycolysis, which phosphorylates hexoses and forms hexose phosphate [11,12]. There are four types of hexokinase isozymes existing in the human biological system. HK2 is widely over-expressed in cancer cells and plays a key role in aerobic glycolysis and anti-apoptosis capacity of tumor cells [13,14]. AKT is a key oncogene protein kinase that controls a variety of cellular functions and has anti-apoptotic effects [15]. Many reports demonstrate that wogonin can inhibit multiple AKT-regulated or -related signaling pathways to induce apoptosis, increase chemotherapy sensitivity, suppress tumor angiogenesis, and so on [16,17,18]. AKT directly phosphorylates Thr-473 of HK2 and promotes the binding of HK2 with voltage-dependent anion-selective channel (VDAC), resulting in the apoptosis resistance [19]. Inactivation of AKT via glycogen synthase kinase-3 beta (GSK-3β) causes disassociation of HK2 from mitochondria. Recent studies show that GL-V9 can induce the lysosome-dependent degradation of AKT1 in T cell malignancies [20], suggesting that GL-V9 might regulate HK2 via AKT-related signaling pathway. Mammalian target of rapamycin (mTOR) is one of the major modulators of autophagy and can be activated by the phosphorylation of AKT [21,22]. Inhibition of AKT/mTOR pathway will damage cell by inducing autophagy [23]. Cell autophagy is associated with proliferation, apoptosis, and metastasis of cancer cells [24]. Autophagy has dual roles in cancer: acting as both a tumor suppressor by preventing the accumulation of damaged proteins and organelles; and as a mechanism of cell survival that can promote the growth of established tumors [25]. Nevertheless, many researchers still focus on the drug development targeting autophagy in cancer, especially potent and specific inhibitors of autophagy.

In this study, we investigated the antineoplastic effect and the underlying mechanism of GL-V9 on cSCC. GL-V9 induced apoptosis as well as autophagy in cSCC via modulation of AKT-related pathway. This research suggests that GL-V9 will be a potential candidate in treatment of cSCC.

## 2. Results

### 2.1. GL-V9 Induces Mitochondria-Mediated Apoptosis of A431 Cells

**Figure 1:**
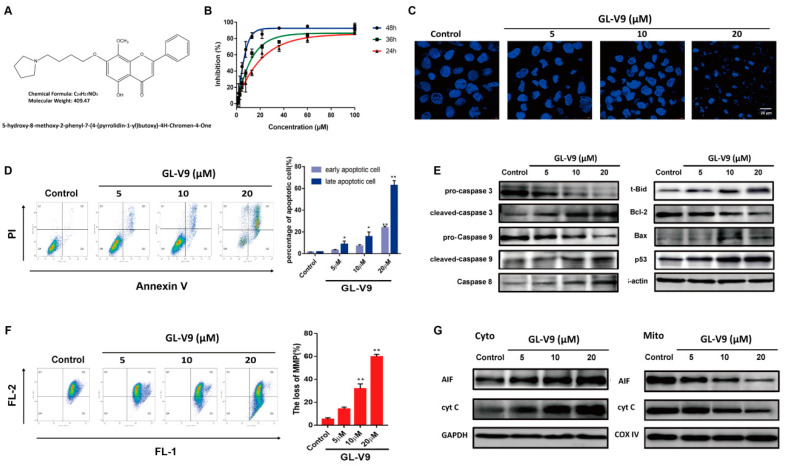
GL-V9 induces apoptosis of A431 cells via mitochondrial mediated pathway. (**A**) The molecular structure of GL-V9 (C_24_H_27_N_5_O, molecular weight 409). (**B**) The inhibitory effect of GL-V9 on A431 cells was detected by MTT assay. (**C**) Nucleolus morphologic changes observed by fluorescent microscope (400×). (**D**) Annexin V/PI double-staining assay by flow cytometry. The apoptotic rates were quantified. (**E**) Western blot assays were used to examine the expression of apoptosis-related proteins. (**F**) Mitochondrial membrane potential (MMP) was determined by flow cytometry. The loss of MMP were quantified. (**G**) The mitochondrial and cytoplasmic extracts were separated. Western blotting was carried out to analyze the expression of Cyt-c, and AIF. Data is shown as average ± SD from three independent experiments. * *p* < 0.05 and ** *p* < 0.01 (comparison to the control 100%).

GL-V9 is a synthetic flavonoid derived from wogonin (Figure 1A). The growth inhibitory effect of GL-V9 in human cSCC cell line A431 cells was firstly measured by MTT assay. Upon 24, 36, and 48 h treatment, GL-V9 inhibited the cell growth of A431 cells in concentration- and time-dependent manners (Figure 1B). IC_50_ values for 24, 36, and 48 h GL-V9 treatment were17.72 ± 4.23, 9.06 ± 0.6, and 5.9 ± 1.14 μM, respectively. Because the concentration-dependence of 36 h GL-V9 treatment was best, we used 5, 10, and 20 μM of GL-V9 treatment for 36 h in the subsequent in vitro studies. Then the roles of GL-V9 in the apoptosis induction was analyzed by DAPI staining and Annexin V/PI double-staining. As shown in Figure 1C, GL-V9 treated A431 cells emitted bright fluorescence, the early phenomena of apoptosis, due to the chromatin agglutination and the nucleolus pyknosis. At the concentration of 20 μM, cellular nucleus of A431 cells disintegrated and formed many nuclear fragments. Annexin V/PI double-staining assay showed that GL-V9 induced apoptosis of A431 cells in a concentration dependent manner (Figure 1D). In further studies, we assayed the expression of some apoptosis-related proteins by western blotting. GL-V9 significantly downregulated the expression of pro-caspase 3, pro-caspase 9, and upregulated the expression of cleaved-caspase 3 and cleaved-caspase 9 (Figure 1E). However, GL-V9 increased protein level of pro-caspase 8 as well as t-Bid. t-Bid, as the actived form of Bid, is cleaved by caspase 8 and induces the release of cytochrome c from mitochondria. Meantime, GL-V9 increased the protein level of p53, downregulated Bcl-2 protein and upregulated bax protein. Bcl-2 and bax were both the downstream gene of p53. All these results suggested that GL-V9 might induce apoptosis via mitochondria-mediated pathway (intrinsic pathway).

The fate of cells succumbing to the intrinsic pathway is triggered by the loss of mitochondrial membrane potential (MMP, ΔΨ_m_), which is an important function index for mitochondria and can be detected by JC-1 probe. As shown in Figure 1F, exposure to GL-V9 for 36 h resulted in the loss of MMP in a concentration dependent manner. We also isolated the mitochondrial and cytoplasmic protein of A431 cell, and investigated the levels of apoptosis-inducing factor (AIF) and cytochrome c in mitochondria and cytoplasm. Western blotting showed that A431 cells treated with GL-V9 for 36 h significantly decreased the level of cytochrome c (cyt-c) and AIF in mitochondria, and increased them in cytoplasm, suggesting GL-V9 promoted the release of cyt-c and AIF from mitochondria (Figure 1G). The results demonstrated that GL-V9 caused mitochondrial dysfunction of A431 cells and induced apoptosis via mitochondrial-mediated pathway.

### 2.2. High Concentration of GL-V9 Induces Autophagy of A431 Cells, Which Potentially Antagonizes Its Apoptosis Inducing Effect

**Figure 2:**
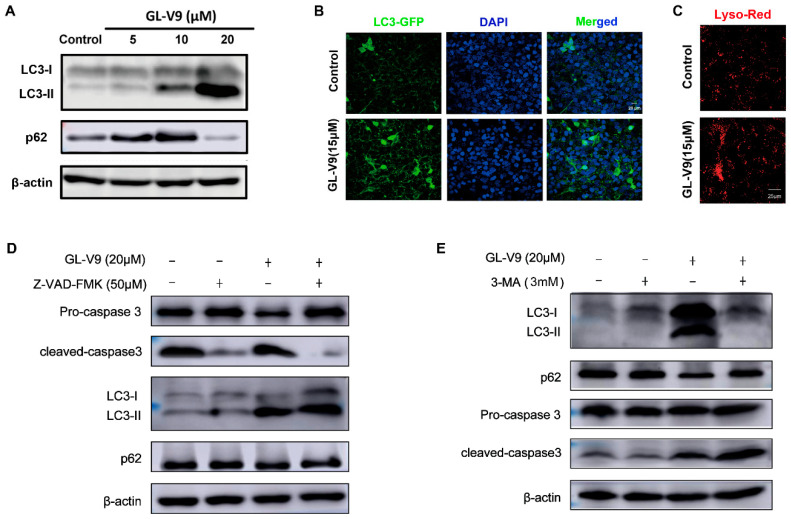
High concentration of GL-V9 induces autophagy of A431 cells, which potentially antagonizes the effect of apoptosis induction. (**A**) Western blot assays were used to examine the expression of autophagy proteins p62 and LC3. (**B**) Immunofluorescence detection of the distribution of GFP-LC3 in A431 cells. Scale bar = 20 μm. (**C**) The lysosome of A431 cells was observed by LysoTracker Red. Scale bar = 25 μm. (**D**) A431 cells were co-treated with GL-V9 with caspase inhibitor Z-VAD-FMK, protein level of caspase 3 and LC3 were assayed. (**E**) A431 cells were co-treated with GL-V9 with autophagy inhibitor 3-MA for 12 h. Then 3-MA was withdrawn and cells were further treated with GL-V9 for another 24 h. Protein level of caspase 3, p62, and LC3 were assayed.

On the other hand, we tested the influence of GL-V9 in the autophagy of A431 cells. As shown in Figure 2A, the soluble form of LC3 (LC3-I) converted to the autophagosome-associated form (LC3-II) upon GL-V9 treatment. The polyubiquitin-binding protein p62 is also used as an autophagic marker, which binds directly to LC3 and is itself degraded by autophagy. Interestingly, low concentration of GL-V9 increased p62 protein level, but high concentration of GL-V9 significantly decreased p62. We thought that the upregulation of p62 by low concentration of GL-V9 might be related with the influence of GL-V9 in energy metabolism. To further confirm the change of autophagy, the GFP-LC3 distribution, extensively used as a biomarker for autophagy, was assayed. As shown Figure 2B, the distribution of GFP-LC3 was changed from largely diffuse to accumulated punctate structures upon GL-V9 treatment by 36 h. Besides, we used LysoTracker Red and found that GL-V9 could promote the aggregation of lysosome of A431 cells (Figure 2C). All the indexes suggested that higher concentration of GL-V9 could induce autophagy of A431 cells.

The connection between autophagy and apoptosis or other forms of cell death is a burgeoning area of research. To investigate whether GL-V9 induced apoptosis and autophagy were synergetic or antagonistic, we used caspase inhibitor Z-VAD-FMK and autophagy inhibitor 3-MA combined with GL-V9. As shown in Figure 2D, Z-VAD-FMK reversed the apoptosis inducing effect of GL-V9, but had little influence in the GL-V9-induced increase of autophagy marker LC3-II. Different with Z-VAD-FMK, after cell autophagy was inhibited by 3-MA, the cleaved of caspase 3 induced by GL-V9 was increased (Figure 2E). These results suggested that the autophagy induced by high concentration of GL-V9 potentially antagonized the effect of apoptosis induction, which might be associated with the influence of cell metabolism. Instead, the overall anti-cSCC effects of GL-V9 were not influenced by the dual regulations of apoptosis and autophagy.

### 2.3. GL-V9 Induced Metabolome Changes in A431 Cells

**Figure 3:**
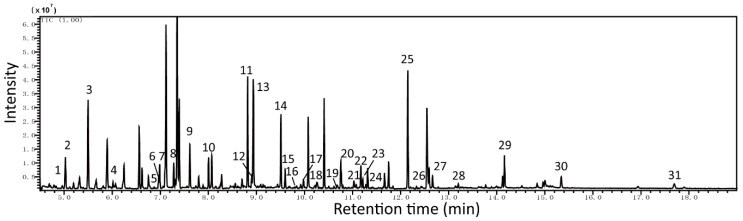
Typical GC/MS chromatograms of extracts from A431 Cell. The metabolites were identified as: 1. Pyruvic acid, 2. Lactic acid, 3. Alanine, 4. Leucine, 5. Serine 6.Isoleucine, 7. Phosphoric acid, 8. Succinic acid, 9. Fumaric acid, 10. Threonine, 11. Malic acid, 12. Aspartic acid, 13. Prolin, 14. Glutamic acid, 15. Phenylalanine, 16. N-Acetylaspartic acid, 17. Asparagine, 18. Tryptamine, 19. Ornithine, 20. 2-Ketoglutaric acid, 21. Cyclopropane, 22. Fructose, 23. Glucose, 24. Tyrosine, 25. Inositol, 26. Ribose-5-phosphate, 27. Sebacic acid, 28. Glucose-6-phosphate 29. Uridine, 30. 5′-Adenylic acid, 31. Cholesterol.

Then we studied the metabolomics of A431 cells treated by GL-V9. The GC/MS analysis of the A431 cells extracts revealed a large number of peaks (Figure 3). Deconvolution of the chromatograms produced a total of 168 distinct peaks and 88 were authentically identified by comparing the mass spectrum of the peak with that available in the libraries and that of the reference metabolites. These included carbohydrates, fatty acids, amino acids, lipids, and amines. To obtain quantitative data, a characteristic mass (*m*/*z*) was selected for each peak, and the peak area was obtained for each inverse volume peak/molecule.

**Figure 4:**
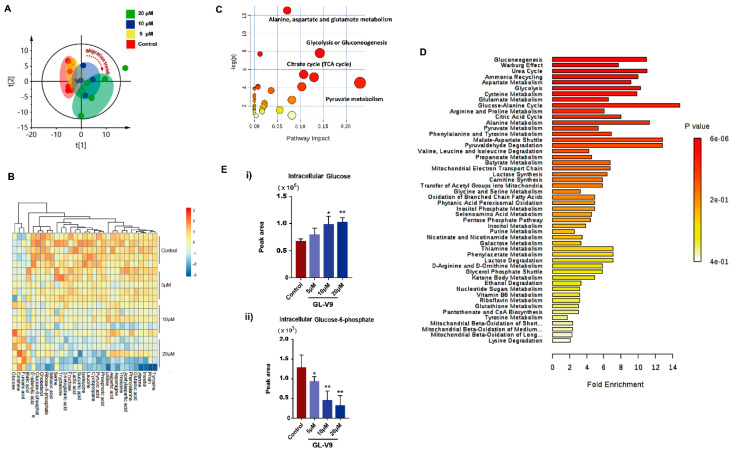
Overview and the pathway of the impact of A431 Cell on metabolites. (**A**) Heatmap of stromal molecular signatures of A431 Cell samples. (**B**) PCA plot of stromal molecular signatures of A431 Cell. Ellipses and shapes show clustering of the samples. (**C**) The pathway impact of A431 Cell on metabolites. The *y*-axis shows the *p*-values and the *x*-axis, the pathway impact values; the node color is based on its *p*-value and the node size reflects the pathway impact values. (**D**) The enrichment overview of the pathway-associated metabolite sets perturbed by acoustic trauma. (**E**) Peak area of glucose and glucose-6-phosphate in intracellular. Data is shown as average ± SD from three independent experiments. * *p* < 0.05 and ** *p* < 0.01 (comparison to the control).

Visual inspection of GC/MS profiles of metabolites in A431 cells of the control group at different GL-V9 concentrations revealed a difference between all administration groups and the control group. Based on the data matrix (with the two vectors of observations/samples and variables/molecules), an unsupervised PCA model was applied to overview the dataset. We found that there was a significant migration trend along with the increase of GL-V9 concentration (Figure 4A). This result suggested that the metabolites and metabolic pathways were changed by GL-V9. In order to more effectively understand the metabolites and metabolic pathways most likely to be changed by GL-V9 in A431 cells, we assayed significant changes of 31 metabolites, involving carbohydrates, amino acids, small organic acids, fatty acids, lipids, and amines. Heatmap were generated for each sample species to visualize the intensities of differential metabolites in different groups (Figure 4B). Further metabolic impact analysis of these selected metabolites showed that GL-V9 had main interference effect on carbohydrate metabolism, tricarboxylic acid cycle (TCA), and pyruvate metabolism (Figure 4C). Enrichment analysis also showed that GL-V9 affected glucose metabolism, Warburg effect, amino acid metabolism, energy metabolism, and mitochondrial function (Figure 4D). According to enrichment analysis, GL-V9 had the greatest influence on glucose metabolism. By investigation of two key substances of glycolysis pathway, glucose and glucose-6-phosphate, we found that intracellular glucose was accumulated but glucose-6-phosphate was decreased upon GL-V9 treatment (Figure 4E). These results suggested that GL-V9 might affect glucose utilization and mitochondrial function of A431 cells. The influence of GL-V9 in the glycolysis resulted in the nutrient deficiency, which may be associated with the increased apoptosis by autophagy inhibitor 3-MA.

### 2.4. GL-V9 Induces Apoptosis by Inhibiting AKT-Regulated Mitochondrial Location of HK2, and Caused Autophagy via Suppression of AKT/mTOR Pathway

**Figure 5:**
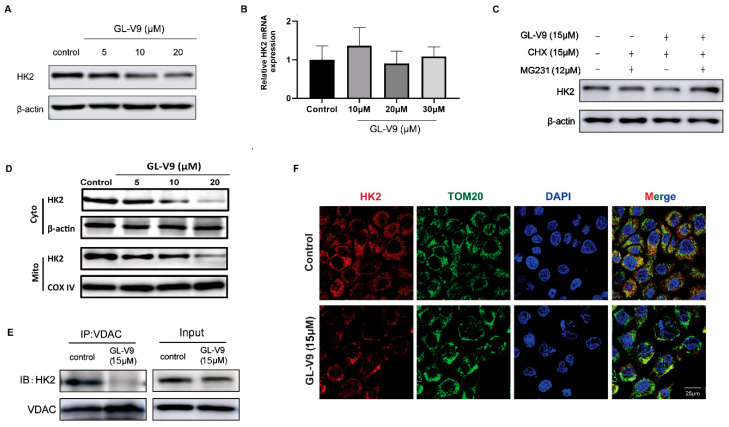
GL-V9 downregulates protein stability of HK2 and inhibits its mitochondrial location. (**A**,**B**) A431 cells were treated with GL-V9 for 36 h. The protein expression (**A**) and mRNA expression (**B**) of HK2 were assayed by western blot and RT-PCR, respectively. Bars, SD from three independent experiments. (**C**) A431 were treated with 15 μM GL-V9 for 24 h, then co-treated with 15μg/mL CHX and 12 μM MG-132 for another 12 h. Then protein level of HK2 was assayed. (**D**) The expression of HK2 proteins in cytosol and mitochondria was examined by western blot. (**E**) The binding of HK2 with VDAC was assayed by immunoprecipitation. (**F**) The location of HK2 in mitochondria was observed by immunofluorescence. TOM20 was a mitochondrial marker. Scale bar = 25 μm.

Hexokinase II (HK2) is dominant isoform of HK in cancer cells, which plays important roles in cell survival and glycolysis [26,27,28]. Because GL-V9 promoted the accumulation of intracellular glucose and decreased glucose-6-phosphate, GL-V9 might influence HK2. We found that GL-V9 downregulated the protein level of HK2, but had no significant influence in its mRNA expression (Figure 5A,B). To investigate that whether GL-V9 influenced the protein stability, we co-treated A431 cells with GL-V9, proteasome inhibitor MG-132 and protein synthesis inhibitor cycloheximide (CHX). As shown in Figure 5C, after CHX blocked the synthesis of HK2, MG-132 reversed the downregulation of HK2 protein by GL-V9. Thus, GL-V9 promoted the protein degradation of HK2 to decrease its level. It is reported that HK2 can bind with VDAC, localize in mitochondria outer membrane and maintain the integrity of mitochondrial structure, leading to the inhibition of apoptosis and promotion of aerobic glycolysis in cancer cells [29]. GL-V9 decreased the level of HK2 in both mitochondria and cytoplasm (Figure 5D). Meanwhile, GL-V9 promoted the dissociation of HK2 with VDCA (Figure 5E). Immunofluorescence assay also showed that the mitochondrial location of HK2 was decreased (Figure 5F).

**Figure 6:**
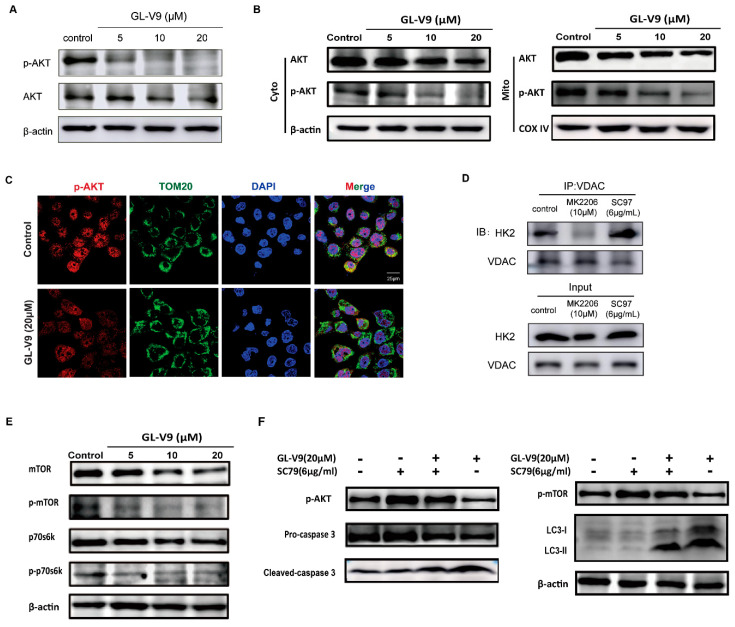
Inhibition of AKT by GL-V9 is responsibility for the decreasing binding of HK2 in mitochondria and induction of autophagy. (**A**) A431 cells were treated with GL-V9 for 36 h. The protein expression of AKT and p-AKT were assayed. (**B**) The expression of AKT and p-AKT proteins in cytosol and mitochondria was examined by western blot. (**C**) The location of p-AKT in mitochondria was observed by immunofluorescence. Scale bar = 25 μm. (**D**) A431 cells were treated with 10 μM MK2206 or 6 μg/mL SC97 for 36 h, respectively. The binding of HK2 with VDAC was assayed by immunoprecipitation. (**E**) Western blot assays were used to examine the protein expression of mTOR, p-mTOR, p70s6k, and p-p70s6k. (**F**) A431 cells were co-treated with SC79 and GL-V9 for 36 h. The expression of key proteins involved with apoptosis and autophagy were assayed.

It is reported that activated AKT (phosphorylated AKT) phosphorylates HK2 and promotes the mitochondrial location of HK2 [30]. We found that GL-V9 downregulated the total level of the AKT and p-AKT as well as their level in mitochondria (Figure 6A,B). The mitochondrial location of p-AKT was decreased as well (Figure 6C). Moreover, AKT inhibitor MK-2206 inhibited the binding of HK2 with VDAC in mitochondria in A431 cells, instead AKT activator SC79 promoted their binding (Figure 6D). These results suggested that GL-V9 inhibited AKT expression and activity, and affected mitochondrial localization of HK2, which was responsible for the mitochondria-mediated apoptosis induced by GL-V9.

AKT/mTOR signaling pathway is one of the classical pathways to regulate autophagy [31]. Therefore, we assayed the influences of GL-V9 in AKT/mTOR pathway in A431 cells. Western blot studies showed that the expression and activation of mTOR were both inhibited by GL-V9 (Figure 6E), because mTOR specifically phosphorylates the p70S6 kinase at Thr-389, the phosphorylation of p70S6 kinase at this position is a routine and specific assay for monitoring mTOR activity [32]. Upon the treatment of GL-V9, the total protein level and the phosphorylation of p70S6K were also reduced (Figure 6E). Thus, GL-V9 induced autophagy via inhibiting AKT/mTOR/p70S6 pathway.

Thus AKT played key roles in the anti-cSCC effect of GL-V9. When A431 cells were co-treated with GL-V9 and AKT activator SC79, the GL-V9-induced changes in caspase 3, p-mTOR, and LC3 were all reversed by SC79 (Figure 6F). All above, GL-V9 induced apoptosis and autophagy of A431 cells by inhibiting the AKT-regulated HK2 and mTOR signals, respectively.

### 2.5. GL-V9 Suppresses the Development of Primary Skin Cancer in Mice

**Figure 7:**
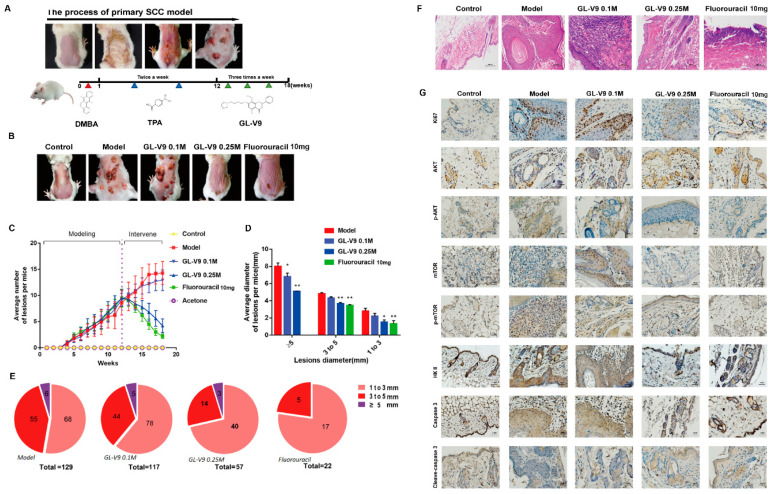
In vivo anticancer effects of GL-V9 on primary skin cancer model. (**A**) Tumor development in DMBA/TPA-induced skin cancer model mice. (**B**) After tumor formation on week 12, mice were applied topically with GL-V9 or 5-Fu to single lesions three times a week. Representative images of papilloma lesion genesis in the indicated groups at week 18. (**C**) The number changes of tumors during the whole process (*n* = 10 per group). Bars, SD. (**D**) Average diameter of lesions per mice (*n* = 10 per group). Bars, SD. * *p* < 0.05 and ** *p* < 0.01 (comparison to the model group). (**E**) Proportion of lesions with different diameters (*n* = 10 per group). (**F**) Images of H&E staining of lesion. Scale bar = 100 μm. (**G**) Proteins expressions in lesion were assessed by immunohistochemistry. Scale bar = 20 μm.

To investigate the in vivo anticancer effect of GL-V9, we performed a mouse two-stage chemical-inducible primary skin cancer model (Figure 7A). In the 18th week of the two-stage chemical-inducible skin cancer model, we can observed cutaneous lesions of papilloma in the back skin of mice. As shown in Figure 7B–E, though the treatment group of low-dose GL-V9 (200 μL of 0.1M GL-V9 solution containing 8.2 mg GL-V9) did not shows a decent anticancer effect, high dose of GL-V9 (200 μL of 0.25M GL-V9 solution containing 20.5 mg GL-V9) had equal effect with fluorouracil (0.2 g of 5% fluorouracil cream containing 10 mg 5-Fu), and could effectively reduce the number and size of cutaneous lesions. The lesions areas skin were cut and used for histological hematoxylin-eosin (HE) staining analysis and immunohistochemistry (IHC) assay. HE staining analysis further indicated that GL-V9 significantly alleviate the increase in hyperplasia caused by two-stage chemical-inducible (Figure 7F). We can observed a large amount of necrotic tissue in the groups with fluorouracil and high-dose of GL-V9 treatment. All these results showed that GL-V9 had potent capacity to suppress the progress of primary skin cancer in mice.

IHC assay was done to further ascertain the effect of GL-V9 on key proteins in vivo (Figure 7G). Ki67, as an important indicator of tumor growth [33]. In the primary skin cancer model group, compared with control group, protein expressions of ki67, AKT, p-AKT, mTOR, p-mTOR, and HKII were all significantly increased. After treatment of high-dose GL-V9 and the fluorouracil, the expression of Ki67, p-AKT, mTOR, p-mTOR, and HK2 were all decreased compared with model group, instead cleaved-caspase 3 were relative increased. These results showed that GL-V9 inhibited the growth of primary skin cancer in mice via suppressing AKT-regulated HK2 and mTOR signals in vivo.

## 3. Discussion

Cutaneous squamous cell carcinoma is an adverse outcome caused by the interaction of many factors, including environmental factors and self-factors [34]. As the elderly population grows and skin cancer screening improving, the incidence rates of cSCC are rising at a rate of 2.5–10% per year, which is becoming a public health problem [35,36]. It is generally accepted that most cSCC can be successfully treated with standard treatments, such as surgical excision, external drug application and local injection therapy. However, a subset of patients are still at risk for local recurrence, peripheral spread, and even metastasis to lymph node or distant organs, especially in individuals with compromised immune function [37]. Imiquimod and 5-Fluorouracil, clinically used for local lesion treatment, usually cause erythema, erosion, and scab, and patients’ compliance [38]. Therefore, it is of great clinical value to develop a novel candidate for cSCC treatment.

**Figure 8:**
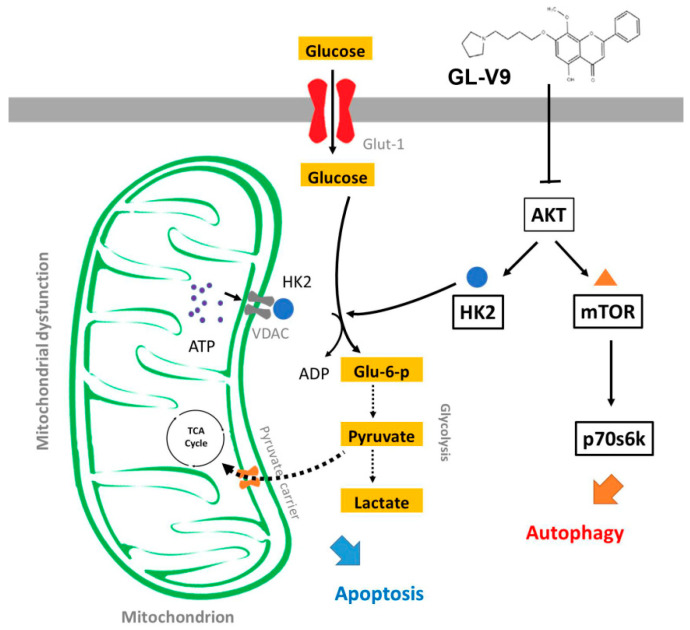
Schematic representation of GL-V9 inducing apoptosis and autophagy via inhibition of AKT-mediated mitochondrial location of HK2 and AKT/mTOR pathway in cSCC.

In this study, we found a synthesized flavonoid GL-V9 derived from natural product wogonin, which had a potent therapeutic effect on cSCC. GL-V9 not only induced apoptosis and autophagy of human cSCC cell line A431 cells, but also suppressed the development of chemical induced primary skin cancer in mice. Besides, GL-V9 caused mitochondrial dysfunction and suppressed glycolysis to reprogram cell metabolism. Mechanism studies showed that AKT played important roles in the anti-cSCC effects of GL-V9, which inhibited AKT-regulated mitochondrial location of HK2 to induce apoptosis, and suppressed AKT/mTOR pathway to activate autophagy (Figure 8).

In recent years, many studies focus on the anticancer effects of the active ingredients of natural products, such as flavonoids, polyphenols, polysaccharide, and so on [39,40,41]. Wogonin, as the precursor of GL-V9, showed obvious pharmacological effects, including anticancer effect. However, the low water-solubility and bioavailability of wogonin limited its development. GL-V9 not only inhibited the cell growth of liver cancer and breast cancer [42,43], also had better solubility and druggability than wogonin [44]. Whereas the effects of GL-V9 in cSCC has not been investigated before. We found that GL-V9 had potent anticancer activity in primary cSCC of mice. High dose of GL-V9 had equal effect with 5-Fu, instead had much lower toxicity than 5-Fu. The toxicity and side effects of 5-Fu greatly limit its therapeutic effect and clinical usage. In addition to some common side effects—including application site reactions (such as redness, burning, erosion pain), alopecia, sinus infection, and so on—fluorouracil cream has severe bone marrow suppression, which will reduce the body’s immune system and greatly increase the death risk. In vivo studies, compared with 5-Fu, none of the mice treated with GL-V9 exhibited any physical discomfiture. Our previous reports also showed the low toxicity of GL-V9 in vivo [45]. No abnormal hematological parameters and morphological changes were observed in the organs of the tumor-bearing mice that were treated with GL- V9. Thus, the development of GL-V9 might provide a potential candidate with less toxicity for cSCC treatment.

AKT promotes the glycolysis and apoptosis via the regulation of mitochondrial HK2 [14,46]. Here, GL-V9 downregulated the protein level and activity of AKT in A431 cells. A previous research have found that GL-V9 induces the lysosome-dependent degradation of AKT1 [20]. Our recent studies also show that GL-V9 may influence the protein stability of AKT by disturbing the binding between Hsp90 and its client proteins (data not shown). The specific mechanism still need further investigation. The suppression of AKT by GL-V9 results in the decrease of mitochondrial HK2. HK2 is highly expressed in various cancers, which binds with the VDAC on the outer mitochondria, enhances aerobic glycolysis and resists apoptosis of cancer cells [27,47]. Differently from normal cells, cancer cells usually tend to choose the aerobic glycolysis to produce energy for cell growth. Metabolism reprograming is considered as a unique characteristic of cancer cells and promising target for cancer treatment [48]. In this study, we analyzed the influences of GL-V9 in the metabolism of A431 cells via metabolomics, and found that GL-V9 reprogrammed the metabolism of cancer cells, mainly influencing glycometabolism, amino acid metabolism, and mitochondrial metabolism. GL-V9 tended to inhibit glycolysis, which might be aroused by suppressing mitochondrial HK2. We could conclude that GL-V9 has significant influence in mitochondrial function of cancer cells. The damage of mitochondria is always accompanied by the changes of intracellular oxidative stress. It has been reported that GL-V9 suppresses thioredoxin-1 and increases reactive oxygen species (ROS) level of hepatoma cells by significantly promoting intracellular O_2_^•−^ level, but not affecting H_2_O_2_ production [7,45]. Although the anticancer efficacy of GL-V9 is better than its precursor wogonin, some reports show that the anticancer effects of GL-V9 is similar to wogonin, such as induction of apoptosis and ROS production [49], and inhibition of PI3K/AKT pathway [50]. However, whether the anti-cancer target of GL-V9 and wogonin are same and has cancer type specificity, still need more investigations. In addition to cell apoptosis, HK2 and AKT are also involved with the regulation of autophagy. It is reported that HK2 prevented autophagy-driven monocyte differentiation [51]. HK2 promotes starvation-induced autophagy by binding to, and inhibiting, TORC1 [52]. Studies have shown that the AKT/mTOR signaling pathway inhibits autophagy death of cancer cells by regulating the transcription and translation of growth promoting oncogenes or proteins [53]. High concentration of GL-V9 inhibits AKT/mTOR pathway, and induces apoptosis as well as autophagy. Recently, the relationship between apoptosis and autophagy are still complicated and confused. A flavonoid named oroxylin A is the isomer of wogonin. It was reported that pharmacological inhibition of autophagy suppressed apoptotic death of HepG2 cells treated with oroxylin A. In this situation, cell apoptosis cooperates with autophagy to promote the death of cancer cells. Instead, inhibition of autophagy by 3-MA promoted the apoptosis induced by GL-V9, which may be explained by the influences of autophagy in apoptotic signals. Some studies report that the clear of apoptosis cell by autophagy is a selective mechanism for cancer cells, which can help cancer cells overcoming the death stress caused by chemotherapy and result in drug resistance [54,55]. Therefore, the further studies of GL-V9 in cancer treatment should take into account of the double effects of apoptosis and autophagy. Co-treatment of autophagy inhibitors or metabolic regulators with GL-V9 will be explored, which may be significantly enhance the therapeutic efficacy.

## 4. Materials and Methods

### 4.1. Agents and Materials

GL-V9 (C_24_H_27_NO_5_, (5-hydroxy-8-methoxy-2-phenyl-7-(4-(pyrrolidin-1-yl)butoxy)4H-chromen-4-one), MW:409.47, 98% purity) was a novel synthetic flavonoid derived from the natural product wogonin and provided by Prof. Zhiyu Li. The synthetic scheme and quality control method are carried out following the previous reports, respectively [8,44]. In vitro experiments, GL-V9 was dissolved in dimethyl sulfoxide (DMSO, Sigma-Aldrich, St. Louis, MO, USA) to the concentration of 0.02 M, stored at −80 °C and diluted to experimental concentrations with DMEM medium (GIBCO, Thermo Fisher Scientific Inc., Chino, CA, USA). SC79 was purchased from MedChemExpress (5 μg/mL, MedChemExpress Co., Ltd., Monmouth Junctioncity, NJ, USA). All antibodies purchased form ABclonal (ABclonal, Wuhan, China). In vivo experiments, GL-V9 was dissolved in acetone solvent that was purchased from TCI and applied to the lesion area, TPA and DMBA were purchased from Sigma Aldrich (Sigma-Aldrich, Co., Ltd., MilliporeSigma, Burlington, MA, USA). Fluorouracil ointment was purchased from EKEAR Bio (5%, EKEAR Bio. Tech., Co., Ltd., Shanghai, China) Proteasome inhibitor MG-132, protein synthesis inhibitor cycloheximide, autophagy inhibitor 3-MA, caspase inhibitor Z-VAD-FMK, AKT inhibitor MK-2206, and AKT activator SC79 were purchased from MCE (MedChemExpress Co., Ltd., Monmouth Junctioncity, NJ, USA). LysoTracker Red was purchased from Keygenbio (KeyGen Biotech Co., Ltd., Nanjing, China).

### 4.2. Cell Culture

Human cSCC cell line A431 cells was purchased from the Cell Bank of Shanghai Institute of Biochemistry and & Cell Biology, Chinese Academy of Sciences. A431 cells were maintained in DMEM medium (GIBCO, Thermo Fisher Scientific CO., Chino, CA, USA) supplemented with 10% Fetal Bovine Serum (FBS, Wisent, Inc., CAN, Quebec, QC, Canada) and 100 U/Ml of streptomycin and penicillin (GIBCO, Thermo Fisher Scientific Inc., Chino, CA, USA). All cells were kept in an incubator at 37 °C in 5% CO_2_ condition.

### 4.3. Animal Model

All mouse experiments were in compliance with policies of the State Food and Drug Administration (SFDA) of China on Animal Care. All animals received humane care according to the criteria outlined in the Guide for the Care and Use of Laboratory Animals prepared by the National Academy of Sciences and published by the National Institutes of Health. Female athymic nude mice (4–6 weeks old) weighing 18–22 g were purchased from the Academy of Military Medical Sciences of the Chinese People’s Liberation Army (certificate no. SCXK (ZHE) 2018–0001). Animals placed in IVC cages with adequate high temperature sterilized water, sterile feed and regular light. The room temperature controlled at 24–26 °C, the humidity was 40–60%. All of the animal experiments are approved by the Institutional Animal Care and Ethics Committee of China Pharmaceutical University. The Ethical Review number is 2020-09-005.

The BALB/c mice (28-days-old, hair follicles were in their anagen, bisexual each half) was firstly shaved off the hair on the back, and used to establish primary skin cancer animal model. Then the mice were administered with DMBA (2,2-Bis(hydroxymethyl)butyric acid) and TPA (12-*O*-tetradecanoyl phorbol-13-acetate) as previously described [56]. Briefly, DMBA (200 μL of 0.3 mg/mL solution in acetone) was applied to shaved skin on the backs of mice in the first week. A week later, TPA (200 μL of 0.04 mg/mL solution in acetone) was then applied to the shaved skin twice a week until 12 weeks when we could observe the cutaneous lesions of papilloma in the back skin.

Then the normal mice were as control, and the tumor-bearing mice were randomly divided into four groups, including model, GL-V9 0.1M, GL-V9 0.25M, and fluorouracil groups (*n* = 10 per group). GL-V9 (dissolved in acetone solvent, 0.25 M and 0.1 M, 200 μL/ per time containing 20.5 mg and 8.2 mg GL-V9, respectively) and fluorouracil Ointment (5%: 10 g, 0.2 g cream/ per time containing 10 mg 5-Fu) were applied to the lesion area three times a week. The choice of GL-V9 dose was referred to the compared growth inhibitory effects of GL-V9 and 5-Fu in mice hepatoma reported previous [57]. During the period of model establishment and drug treatment, the mice of control group were applied with same volume of acetone on the back skin, parallelly. In 18 weeks, all mice were killed and their back skin was taken for hematoxylin–eosin and immunohistochemical staining testing.

### 4.4. MTT Assay

A431 cells were treated with 1.0–100 μM GL-V9 for 24, 36, and 48 h, respectively. After incubation, 20 μL of 5 mg/mL MTT was added. Four hours later, the culture medium was removed and the formed formazan was dissolved by 100 μL DMSO per well. The absorbance at 570 nm was measured by Universal Microplate Reader (EL800, BIO-TEK Instruments Inc., Winooski, VT, USA).

Cell Inhibition ratio (*I%*) = (*A_control_* − *A_treated_*)/*A_control_* × 100% (1)

where *A_control_* and *A_treated_* are the average absorbance of three parallel experiments from treated and control groups, respectively. The IC_50_ was taken as the concentration that caused 50% inhibition of cell proliferation. To determine the IC_50_ value, we fit the data using an equation for a sigmoidal dose-response provided by GraphPad™ Prism^®^ 4.0.

### 4.5. Apoptosis Assay

A431 cells were treated with GL-V9 for 36 h and harvested. Apoptotic cells were identified by double supravital staining with recombinant FITC (fluorescein isothiocyanate)-conjugated Annexin- V and PI, using the Annexin V–FITC Apoptosis Detection kit (BioVision, Inc., Milpitas, CA, USA) according to the manufacturer’s instructions. Flow cytometric analysis was performed immediately after supravital staining. Data acquisition and analysis were performed in a Becton–Dickinson FACSCalibur flow cytometer using Cell Quest software Pro V5.2.

### 4.6. DAPI Staining

Administrated A431 cells were seeded on to glass coverslips processed for immunofluorescence. The glass coverslips were washed twice with cold PBS for 5 min, fixed with 4% paraformaldehyde (PFA) for 30 min, and incubated with Triton X-100 for 10 min. After incubation, the A431 cells were blocked with PBS containing 3% BSA for 1 h, and then the coverslips were stained with diamidino-phenyl-indole (DAPI, purchased from Beyotime Biotechnology Co., Hangzhou, China) for 30 min. The images were captured with an Olympus FV1000 confocal microscope (Olympus Corp., Tokyo, Japan).

### 4.7. Mitochondrial Membrane Potential (MMP) Assay

The loss of MMP was measured by the Mitochondrial Membrane Potential Detection kit (KeyGen Biotech Co., Ltd., Nanjing, China). After being incubated with GL-V9 for 36 h, all floating and attached cells were harvested and resuspended with ice-cold PBS (2000 rpm × 5 min). Then the cell suspensions were incubated in JC-1 prepared with 1× Incubation Buffer for 20 min at 37 °C, and detected by FACSCalibur flow cytometry (Becton Dickinson, Franklin Lakes, NJ, USA).

### 4.8. Extraction of Mitochondrial and Cytosolic Fractions

The mitochondrial and cytosolic fractions of A431 cells were performed using mitochondria/cytosol fractionation kit (KeyGen Biotech, Nanjing, China) according to the following protocol. The A431 cells were administrated with different concentrations of GL-V9 for 36 h and were collected and keep warm in 100 mL ice-cold mitochondrial lyses buffer for 10 min. The suspension of A431 cell was homogenized for strike with a tight pestle. The homogenate was subjected to centrifuging at 600× *g* for 10 min at 4 °C to remove nuclei and unbroken cells. Then the collection was harvested and centrifuged again at 12,000× *g* for 30 min at 4 °C to obtain the supernatant of cytosol and deposition of mitochondria fraction. Samples of cytosol and mitochondria were dissolved in lyses buffer at −20 °C.

### 4.9. Immunofluorescence

Cells were collected and seeded onto glass coverslips processed for immunofluorescence. The glass coverslips were washed twice with cold PBS for 5 min, fixed with 4% paraformaldehyde for 20 min and incubated with 0.2% Triton X-100 for 5 min at 4 °C. After incubation, the cells were blocked with PBS containing 3% BSA for 1 h and incubated with anti-HK2 antibody (1:100, abclonal), anti-p-AKT antibody (1:100, abclonal) as well as anti-TOM20 antibody (1:400, abcam) overnight. After being washed twice with cold PBS for 10 min, the cells were stained with FITC-conjugated Goat Anti-Rabbit and PE-conjugated Goat Anti-Mouse IgG second antibody (1:500, abcam) for 1 h. The images were captured with a confocal microscope (Olympus FV1000, Olympus Corp., Tokyo, Japan).

### 4.10. Immunoprecipitation

For co-immunoprecipitation of VDAC-HK2 complexes, VDAC were immunocaptured from mitochondrial extracts. Lysate of mitochondrial fraction (1 mL) containing 1.5 mg total protein was incubated, respectively, with 1 mg VDAC antibody and 20 mL protein A/G-conjugated beads (Santa Cruz Biotechnology, Santa Cruz, CA, USA) overnight. After four washes with TNES buffer, samples were centrifuged at 3000× *g* for 2 min and resuspended in 20 mL SDS-sample buffer (0.5 M Tris–HCl, pH 6.8, 20% glycerol, 2% SDS, 5% 2-mercaptoethanol, 4% bromophenol blue). For western blot analysis, 10 μL samples were used. The immunocomplexes were analyzed by western blotting and probed with antibody against ant HK2 and VDAC antibody.

### 4.11. Real-Time PCR Analysis

Total RNA was extracted using TriPure Isolation Reagent (Roche Diagnostics, Mannheim, Germany). One microgram of total RNA was used to transcribe the first strand cDNA with SuperScript II reverse transcriptase (Invitrogen). Real-time PCR was completed on an ABI PRISM Sequence Detector 7500 (PerkinElmer, Branchburg, NJ, USA) using Sequence Detector version 1.7 software (Applied Biosystems, Foster City, CA, USA). SYBR Green PCR Master Mix was purchased from Applied Biosystems. Forward and reverse primers for targeted mRNA were designed and purchased from TAKARA BiotechnologyCo., Ltd. (Dalian, China). The primer sets used in the PCR assay were as follows:

HK2-sense: 5′-GGCTCTGGACAGGTGGTAAAGA-3′;

HK2-antisense: 5′-CGGTAATGCACCACCTTGGTGT-3′;

GAPDH -sense: 5′- TAGTGGAAGGACTCATGACC-3′;

GAPDH-antisense: 5′-TCCACCACCCTGTTGCTGTA-3′

Fold change of mRNA level was calculated. After completion of the PCR, the baselines and thresholds were set for both samples and internal GAPDH control.

### 4.12. Western Blotting

Used lysis buffer isolated Proteins, incubated in SDS buffer, separated on SDS-polyacrylamide gels and electroblotted onto PVDF (poly vinylidene fluoride) membranes. Immunoreactivity protein bands were detected using an Amersham Imager 600 RGB (GE Healthcare, Chicago, IL, USA). The following antibodies were used for western blotting: caspase-3, cleaved caspase-3, caspase-8, caspase-9, cleaved caspase-9 at 1:1000 dilution; and bcl-2, bax, t-bid, p53, cytochrome C, AIF, HK2, P62, LC3, and AKT-mTOR (ATK, p-AKT, mTOR, p-mTOR, p70, p-p70) signaling pathway proteins at 1:1200 dilution; GAPDH, COX IV, and β-actin at 1 : 2000 dilution.

### 4.13. Immunohistochemical Staining

For IHC analysis, mice skin was collected and paraformaldehyde fixed, paraffin-embedded sections of skin tissues (4 μm thick) were mounted on slides coated with 2-aminopropyltriethoxysilane, which then were baked, deparaffinized, rinsed with 3% hydrogen peroxide, and incubated with proteinase K (0.5 mg/mL). After that, these sections were washed and then blocked with StartingBlockTM blocking buffers (Pierce, Rockford, IL, USA) for 5 min and subsequently incubated with corresponding polyclonal antibodies for 30 min. Finally, the sections were incubated with Strept Avidin-Biotin Complex (Solarbio) for 30 min at room temperature, followed by detection with a 3,3-diaminobenzidine tetrahydrochoride solution (chromogen) (ZSGB-BIO) and hematoxylin (counterstain). Sections were further mounted with neutral gums. IHC sections were photographed by Mantra 1.01 (PerkinElmer, Waltham, MA, USA).

### 4.14. Metabolomics

For metabolomics analysis, cells and medium samples were pretreated, extracted, and derivatized as previously reported [57]. Chromatographic separation of the analysts was achieved with in Shimadzu GCMSQP2010 (Shimadzu Corp., Tokyo, Japan) equipped with a RTx-5MS column (30 mm × 0.25 mm i.d. fused-silica capillary column chemically bond with a 0.25 μm cross bond, 5% diphenyl/ 95% dimethyl polysiloxane, Restek Corporation, PA, USA). The raw data acquired with GC/MS were processed, and the metabolites were identified. After identification, to selected one feature ion as the quant mass, and the peak area was acquired for each peak, respectively. In addition, metabolic pathway and enrichment analysis were performed by inputting discriminant molecules into Metaboanalyst 4.0.

### 4.15. Multivariate Statistical Analysis

The relative quantitative data for each peak (peak area) was first normalized against the IS, and then the data matrix was constructed by the normalized peak areas with the sample names as observations in the first column, and retention times/peaks as the response variables in the first row. The heatmap was assessed using the R language with own coded. The multivariate data was evaluated using the SIMCA P 13.0 software (Umetrics, Umeå, Sweden).

### 4.16. Transient Tranfection

The green fluorescent protein (GFP) and microtubule associated protein 1 light chain 3 (LC3) fusion plasmid pcDNA-GFP-LC3 was purchased from BioVector (Biovector Science Lab, Inc., Peking, China). For transfection, cells were seeded in 6-well plates at a density of 5000 cells/well. Plasmids were introduced 2 μg/well into the cells using Lipofectamine™ 2000 reagent (Invitrogen, Thermo Fisher Scientific Inc., Chino, CA, USA) according to the manufacturer’s recommendations.

### 4.17. Statistical Analysis

Values were expressed as the mean ± SD of at least three independent experiments. One way analysis of variance (ANOVA) was used to compare in groups.

## 5. Conclusions

Collectively, our findings demonstrate that the GL-V9 is a potent candidate for the treatment of local cutaneous squamous cell carcinoma via induction of apoptosis and autophagy. Inhibition of AKT plays important roles in the anti-cSCC effects of GL-V9, which leads to the suppression of AKT-mediated HK2 location in mitochondrial and AKT/mTOR pathway. The findings that GL-V9 has double regulation in apoptosis and autophagy will provide some insights for the therapeutic strategies of cSCC.
